# Polarized subsets of human T-helper cells induce distinct patterns of chemokine production by normal and systemic sclerosis dermal fibroblasts

**DOI:** 10.1186/ar1860

**Published:** 2005-11-30

**Authors:** Carlo Chizzolini, Yann Parel, Agneta Scheja, Jean-Michel Dayer

**Affiliations:** 1 Immunology and Allergy, Geneva University Hospital, Geneva School of Medicine, Rue Micheli-du-Crest, 24, 1211 Geneva 14, Switzerland; 2 Division of Rheumatology, Lund University Hospital, 221 85 Lund, Sweden

## Abstract

The role of fibroblasts in inflammatory processes and their cross-talk with T cells is increasingly being recognized. Our aim was to explore the capacity of dermal fibroblasts to produce inflammatory chemokines potentially involved in fibrosis occurring in response to contact with polarized human T cells. Our findings indicate that the program of chemokine production by fibroblasts is differentially regulated depending on the T-helper (Th) cell subset used to activate them. Thus, Th1 and Th2 cells preferentially induced production of IFN-γ inducible protein (IP)-10 and IL-8, respectively, whereas monocyte chemoattractant protein (MCP)-1 was equally induced by both subsets at mRNA and protein levels. Neutralization experiments indicated that membrane-associated tumour necrosis factor-α and IL-1 played a major role in the induction of IL-8 and MCP-1 by Th1 and Th2 cells, whereas membrane-associated IFN-γ (present only in Th1 cells) was responsible, at least in part, for the lower IL-8 and higher IP-10 production induced by Th1 cells. The contributions of tumour necrosis factor-α, IL-1 and IFN-α were confirmed when fibroblasts were cultured separated in a semipermeable membrane from living T cells activated by CD3 cross-linking. We observed further differences when we explored signal transduction pathway usage in fibroblasts. Pharmacological inhibition of c-Jun N-terminal kinase and nuclear factor-κB resulted in inhibition of IL-8 mRNA transcription induced by Th1 cells but not that by Th2 cells, whereas inhibition of MEK/ERK (mitogen-activated protein kinase of extracellular signal-regulated kinase/extracellular signal-regulated kinase) and nuclear factor-κB resulted in inhibition of MCP-1 mRNA induced by Th2 but not by Th1 cells. Finally, no distinct differences in chemokine production were observed when the responses to T cell contact or to prototypic Th1 and Th2 cytokines were examined in systemic sclerosis versus normal fibroblasts. These findings indicate that fibroblasts have the potential to participate in shaping the inflammatory response through the activation of flexible programs of chemokine production that depend on the Th subset eliciting their response.

## Introduction

Fibroblasts are cells of mesenchymal origin and are principally involved in the generation and maintenance of extracellular matrix. Fibroblast morphology, phenotype and function may vary depending on the tissue of origin and on whether the tissue is exposed to physiological or pathological conditions. Thus, cultured fibroblasts derived from skin, breast, lung and haematopoietic tissue have been shown to express structural, extracellular matrix and surface proteins differentially, and to produce different cytokines [1-3]. Chemokine production may also vary depending on the source of fibroblasts, and differences in the levels of eotaxin/CC chemokine ligand (CCL)11, IL-8/CXC chemokine ligand (CXCL)8, monocyte chemoattractant protein (MCP)-1/CCL2, RANTES (regulated upon activation normal T cell expressed and secreted)/CCL5, and macrophage inflammatory protein (MIP)-1α/CCL3 have been reported [3]. In addition, production by fibroblasts of chemokines may be variably modulated by cytokines, with differences being related to the origin of the fibroblasts [4-8].

Chemokines are soluble mediators that were originally identified because of their chemotactic properties in cells expressing specific receptors. Indeed, chemokines that influence chemotaxis regulate leucocyte homeostasis and recruitment of leucocyte subpopulations at sites of inflammation [9]. However, their biological functions are broader, comprising relevant roles in virus cell entry, angiogenesis, tumour growth, metastasis formation and fibrosis [10]. For instance, MCP-1/CCL2 – a CC chemokine that binds to CC chemokine receptor (CCR)2 – has attracted keen interest in the field of fibrosis because it appears to play direct roles in collagen and matrix metalloproteinase-1 induction on fibroblasts [11-13] and is present at sites undergoing fibrosis. In human systemic sclerosis (SSc), MCP-1 mRNA proved to be the most abundant mRNA when bronchoalveolar lavage cells from SSc lung were compared with controls using microarray technology and testing a total of 4507 genes [14]. Moreover, it is produced in large amounts by SSc skin fibroblasts [13,15,16]. Of interest, IL-4 triggers MCP-1 production by human lung fibroblasts [17], and MCP-1 may polarize T cells toward a T-helper (Th)2 subset in mouse [18,19]. In a rodent model of fibrotic versus nonfibrotic pulmonary granulomas, procollagen production was associated with Th2 cells and MCP-1 production [20]. Furthermore, mice null for CCR2 were resistant to development of lung fibrosis induced by transgenic IL-13 [21] and bleomycin [22].

Several additional chemokines have been detected by histological or molecular biological methods at sites undergoing fibrosis in humans or mouse models, including the CC chemokines RANTES [23], MIP-1α [24], PARC (pulmonary and activation-regulated chemokine)/CCL18 [25] and MCP-3/CCL7 [26], and CXC chemokines IL-8/CXCL8, GRO (growth regulated oncogene)-α/CXCL1 [27], ENA-78 (neutrophil-activating peptide-78)/CXCL5 and MIP-2 [28]. With the exception of PARC [25], it is not known whether these chemokines play direct profibrotic or antifibrotic activities apart from recruiting specific leucocyte subsets [3]. Nonetheless, it has been suggested that the proangiogenic and antiangiogenic activities of chemokines play important roles in fibrosis [29]. In bleomycin-induced lung fibrosis, neutralization of MIP-2 (a possible murine analogue of human IL-8) attenuates fibrosis [28], and systemic administration of IFN-γ inducible protein (IP)-10 or transgenic overexpression of IP-10 reduces fibrosis [30,31].

SSc is a human disease that is presumably of autoimmune origin and is characterized by vasculopathy and fibrosis of the skin and internal organs. In the early stage of the disease, inflammatory infiltrates rich in T cells dominate in tissues undergoing fibrosis, and fibroblasts adjacent to T cells exhibit high metabolic activity (for review, see the report by Chizzolini [32]). T cells infiltrating the skin or recovered from bronchoalveolar lavage fluid from SSc individuals predominantly express the Th2 phenotype [33-36], which is consistent with a profibrotic activity of Th2 cytokines [37-39]. In addition, we addressed the ability of T cells to regulate extracellular matrix deposition by cell–cell interaction with fibroblasts [36,40]. However, no data exist on the capacity of T cells to elicit the production of chemokines by dermal fibroblasts and, in particular, the capacity of polarized T cells (Th1 and Th2) to modulate fibroblast production of chemokines differentially. Thus, we conducted the present study to assess whether polarized human T cells could, in a contact-dependent manner, induce dermal fibroblasts to produce MCP-1, IL-8 and IP-10. We opted to focus on these chemokines because of their potential role in the development of fibrosis, particularly in SSc.

## Materials and methods

### Patients and control individuals

A skin punch biopsy, 3 mm in diameter, was obtained from areas of affected skin from eight SSc patients at the Division of Rheumatology in Lund (Sweden) and from eight age-matched and sex-matched healthy control individuals. All SSc individuals fulfilled the American College of Rheumatology criteria for SSc [41] and had clinical features of early disease, and none was undergoing immunosuppressive therapy [42]. Permission to perform this investigation was granted by the ethics committee. Informed consent was obtained from all individuals. Peripheral blood from healthy individuals was provided by the Blood Transfusion Center of the Geneva University Hospital (Switzerland).

### Reagents

Anti-CD3 OKT3 mAb and anti-human IL-4 mAb (clone 25D2) were from ATCC (Bethesda, MD, USA). Anti-human IFN-γ mAb was a gift from Dr G Garotta (Serono Biomedical Research Institute, Geneva, Switzerland). IgG_1 _mAb (anti-TNP, clone G106HN) was a kind gift from S Izui (Department of Pathology and Immunology, Geneva School of Medicine, Geneva, Switzerland). Anti-CD40L (mAb 5c8) was a kind gift from P Lipski (University of Texas, Dallas, TX, USA). Human recombinant (r)IL-4 was from Schering Plough (Dardilly, France). Human rIL-2 was from Biogen (Cambridge, MA, USA). Human rIL-13 was from Sanofi (Montpellier, France). Human rIFN-γ was from Roussel Uclaf (Paris, France). Human recombinant transforming growth factor (rTGF)-β was from R&D (Minneapolis, MN, USA). Anti-TNF (recombinant-methionyl soluble TNF-type I pegylated receptor [sTNF-RI]) [43] and recombinant human IL-1 receptor antagonist [44] were from Amgen (Thousand Oaks, CA, USA). Recombinant human CD40L trimer/leucine zipper fusion protein was from Immunex (Seattle, WA, USA) [45]. RPMI-1640, Dulbecco's modified Eagle medium (DMEM), glutamine, penicillin, streptomycin, trypsin, nonessential amino acids, sodium pyruvate and foetal calf serum (FCS) were from Gibco (Paisley, Scotland). Sucrose, phenyl methyl sulfonyl fluoride, pepstatin, EDTA, iodoacetamide, NP40 and indomethacin were from Sigma (St. Louis, MO, USA). Proteasome inhibitor I (PSI; Z-Ile-Glu [O*t*Bu]-Ala-Leu-CHO), c-Jun NH_2_-terminal kinase (JNK) inhibitor SP600125 (anthra [1,9-cd]pyrazol-6(2H)-one), and mitogen-activated protein kinase of extracellular signal-regulated kinase (MEK)1/2 inhibitor U-0126 (1,4-diamino-2,3-dicyano-1,4-*bis *[2-aminophenithiol]butadiene) were from Calbiochem (San Diego, CA, USA).

### T cell clones and T cell membrane preparation

Prototypic Th1 and Th2 cell clones were generated from peripheral blood of normal individuals upon antigen activation and cloning by limiting dilution in RPMI-1640 medium supplemented with IL-2 (20 U/ml), penicillin (50 U/ml), streptomycin (50 μg/ml), 5% human AB serum, 10% FCS, and irradiated (3,500 rads) allogeneic peripheral blood mononuclear cells with phytohaemagglutinin (1 μg/ml) [46]. Growing cells were further expanded and characterized with respect to their capacity to produce IFN-γ and IL-4 upon CD3 crosslinking. High IFN-γ/low IL-4 producers were defined as Th1, whereas low IFN-γ/high IL-4 producers were defined as Th2. In addition, we used SSc skin-derived polarized T cell clones (generation and characterization of which are described elsewhere [36]). For the preparation of T cell membranes, T cells (8 × 10^6^) were activated in six-well trays, with plastic-adsorbed anti-CD3 mAb. Controls consisted of T cells cultured in medium alone. After six hours of culture the supernatants were collected and frozen for further cytokine determination, and cell membranes were prepared as described elsewhere [46].

### Skin fibroblast–lymphocyte cocultures

Fibroblasts from skin biopsy were grown in DMEM medium supplemented with 10% FCS, penicillin, streptomycin, nonessential amino acids and sodium pyruvate, and split at confluence. All of the experiments were performed with fibroblasts at passages four to nine. In order to study chemokine production, we plated fibroblasts in 96-well trays at 2 × 10^4 ^cells/well in 200 μl DMEM supplemented with 10% FCS, which were then cultured for 72 hours. The culture medium was then replaced by DMEM supplemented with 1% FCS. To assess the effect of T cell contact on chemokine production, 5 μl of T cell membranes equivalent to 2 × 10^5 ^cells/well, unless otherwise stated, was added to fibroblasts in triplicate wells and cultured for 48 hours. In no instance were T cells syngeneic with fibroblasts. Supernatant was then harvested and frozen until chemokine determination. In blocking experiments anti-TNF (soluble TNF receptor I; 10^-8 ^mol/l), anti-IFN-γ (10 μg/ml) [40], IL-1 receptor antagonist (2 μg/ml) [44], anti-IL-4 (10 μg/ml) and irrelevant monoclonal IgG_1 _(10 μg/ml), alone or in combination, were added to the wells 30 minutes before T cell membranes [47,48]. To evaluate the effect of cytokines on chemokine production by fibroblasts, TGF-β (10 ng/ml), IL-4 (10 ng/ml), IL-13 (10 ng/ml) and IFN-γ (1000 U/ml) were added to triplicate wells. When used in combination, TGF-β, IL-4 and IL-13 were used at 5 ng/ml each. In some experiments 20 × 10^3 ^fibroblasts were cultured in the upper chamber of a semipermeable polyester membrane transwell and 10^6 ^living T cells were cultured in the lower chamber, in which anti-CD3 mAb had been plastic adsorbed (Transwell #347-clear; Costar, Corning, NY, USA) for 48 hours. The total culture volume was 1 ml.

### RNA extraction and RNase protection assay analysis

Fibroblasts (5 × 10^5^) were plated to confluence in 100 mm Petri dishes and serum starved overnight. T cell membranes to the equivalent of 8 × 10^6 ^cells were then added to the cultures (typically 200 μl of cell membranes in 3 ml) and fibroblasts were cultured for four additional hours in 1% FCS medium. When used, intracellular signalling inhibitors (PSI [40 μmol/l], JNK-inhibitor [10, 20, and 50 μmol/l], U-0126 [40 μmol/l]) were added 1 hour before adding T cell membranes. Total fibroblast RNA was isolated with TRIzol™ reagent (Life Technologies, Invitrogen, Carlsbad, CA, USA). The levels of expression of IL-8, MCP-1, IP-10 and L-32 mRNAs were assessed by RiboQuant RPA using the hCK-5 multiprobe template set from Pharmingen (San Diego, CA, USA), in accordance with the supplier's instructions. After phosphor imaging (Typhoon 9400, Applied Byosistems, Foster City, CA, USA), signal intensity was determined by densitometry using ImageQuant software (Molecular Dynamics, NIH, Bethesda, MD, USA), and normalized values were used to determine the effect of T cells and signal inhibitors.

### Cytokine determination

Levels of IL-8, MCP-1, IP-10, IL-4, IFN-γ and TNF-α (R&D Systems, Abingdon, UK), and IL-1α (Immunotech, Marseille, France) proteins were assessed using commercial enzyme-linked immunosorbent assay. T cell membranes were solubilized in 1% NP40 to detect cytokine content.

### Statistical analysis

Student's *t *test was used with two-tailed *P *values or Mann–Whitney *U *test using StatView 5.0™ (SAS Institute Inc., Cary, NC) software on a Macintosh computer. *P *< 0.05 was considered statistically significant.

## Results

### Chemokine production by fibroblasts in response to T cell contact

**Figure 1:**
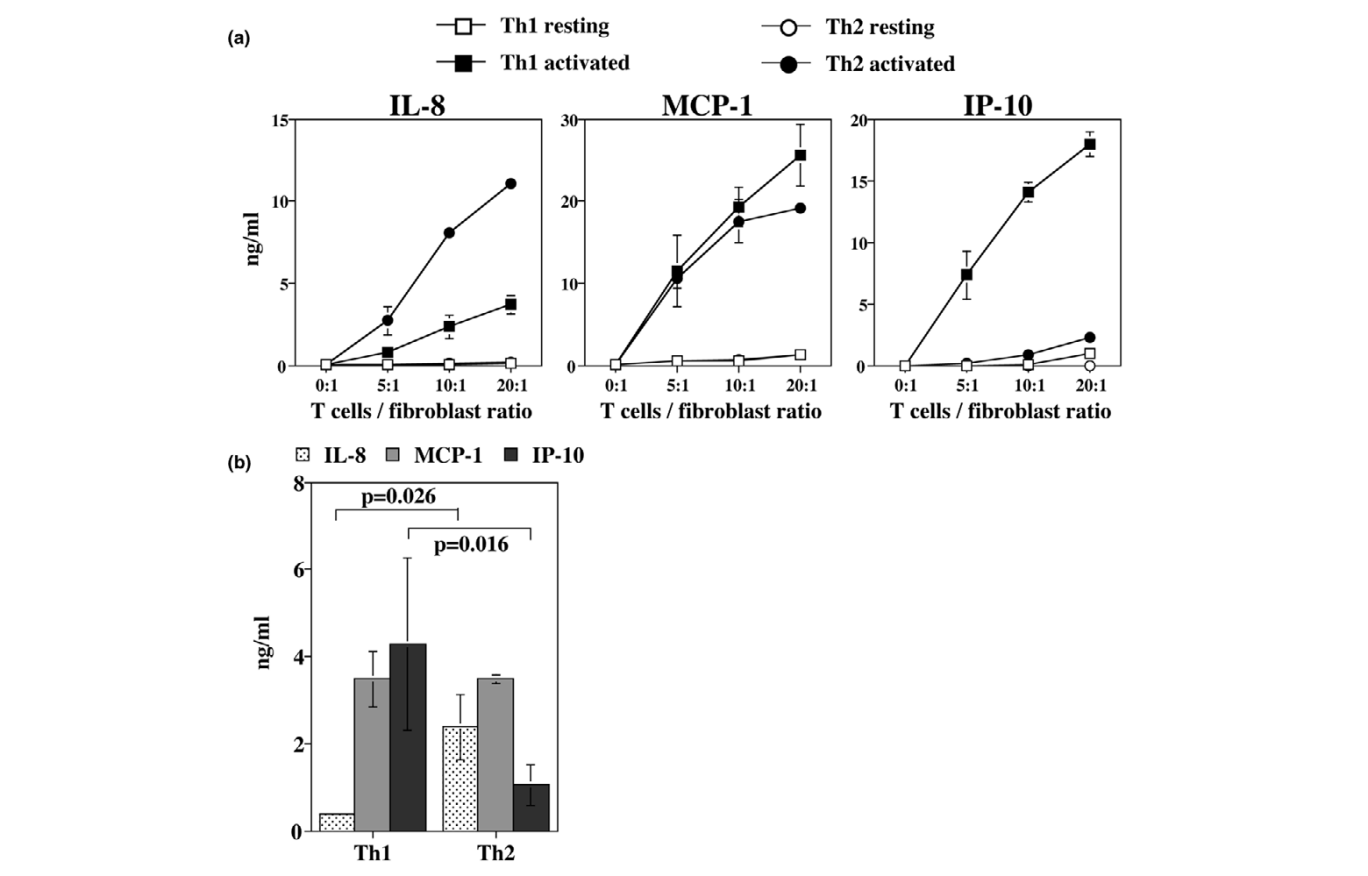
IL-8, MCP-1 and IP-10 production by dermal fibroblasts following contact with T-helper cells. Normal fibroblasts were plated at 2 × 10^4 ^cells/well; 72 hours later the culture medium was replaced and T cell membranes generated from resting or activated Th1 and Th2 clones were added to the wells to a final volume of 200 μl. Chemokine levels were determined in the supernatants by enzyme-linked immunosorbent assay after 48 hours of further culture. **(a) **The points represent the mean ± standard deviation of triplicate cultures. Similar results were obtained in an additional experiment. **(b) **T cell membranes corresponding to 2 × 10^5 ^cells generated from activated Th1 (*n *= 10) and Th2 (*n *= 10) clones were added to the wells in triplicate cultures. The bars represent the mean ± standard error. IL, interleukin; IP, interferon-γ inducible protein; MCP, monocyte chemoattractant protein; Th, T-helper.

We first tested whether human dermal fibroblasts were able to produce IL-8, MCP-1 and IP-10 when activated in a contact-dependent manner by polarized T cell clones [36]. To minimize the effect of soluble mediators, we used T cell membranes as effectors of contact-dependent activation. Upon T cell contact, dermal fibroblasts produced IL-8, MCP-1 and IP-10, with the production depending on whether T cells were resting or activated (CD3 crosslinking) and on the subset of Th cells (Figure 1a). To induce chemokine production, both Th1 and Th2 cells had to be activated because chemokine production was marginal in the presence of resting T cells (Figure 1a). Dose-dependent increases in IL-8, MCP-1 and IP-10 were observed in the presence of activated T cells. However, Th1 cells were less potent inducers of IL-8 and stronger inducers of IP-10 than were Th2 cells, although both subsets potently induced MCP-1 (Figure 1a).

We extended these findings by testing a large panel of polarized T cell clones at a fixed ratio of T cells to fibroblasts of 10:1. Under these settings, Th1 cells induced statistically significantly higher amounts of IP-10 than did Th2 cells, and Th2 induced higher amounts of IL-8 than did Th1 cells, with no differences being observed in MCP-1 levels (Figure 1b). Of note, the levels of IP-10 produced by fibroblasts were positively correlated (R_2 _= 0.813) with the potential for IFN-γ production by T cell clones. No correlation was observed with the levels of the other chemokines, or were chemokine levels correlated with the levels of IL-4.

Finally, we tested whether differences existed between dermal fibroblasts generated from normal skin and those from SSc skin. Although no differences were observed in response to T cell contact between control and SSc fibroblasts, differences in the levels of cytokines were striking depending on the origin of T cells, with Th1 cells being more potent inducers of IP-10 and Th2 being stronger inducers of IL-8 (data not shown). These findings indicate that dermal fibroblasts upregulate their production of chemokines when activated by T cells and that the type of T cell dictates which chemokine is preferentially produced.

### Chemokine production by fibroblasts in response to T cell cytokines and TGF-β

**Figure 2:**
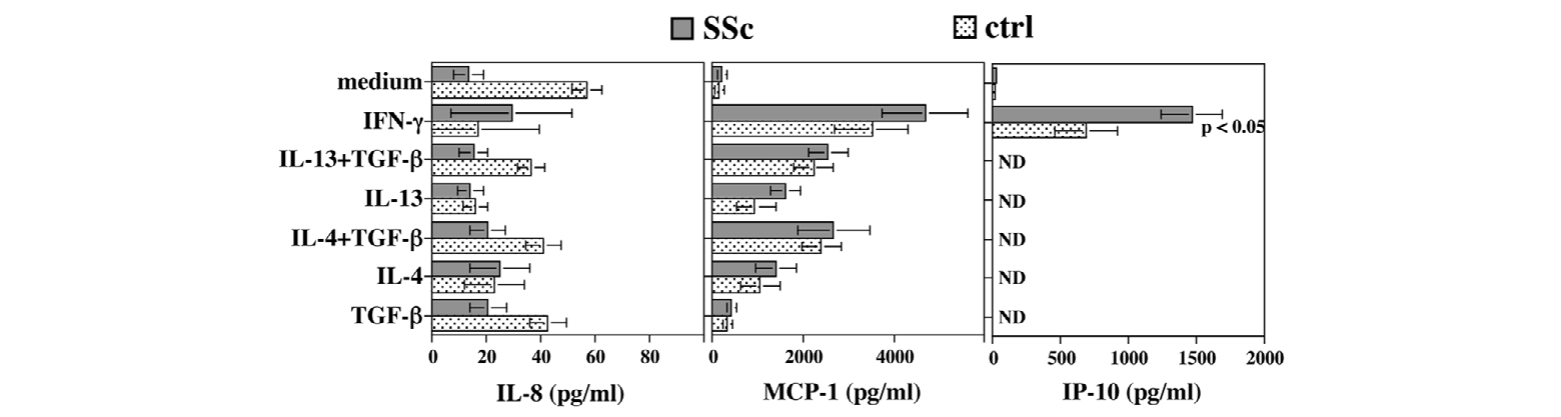
IL-8, MCP-1, and IP-10 production by SSc/control fibroblasts in response to TGF-β and T cell cytokines. Fibroblasts were plated at 2 × 10^4 ^cells/well; 72 hours later the culture medium was replaced and TGF-β (10 ng/ml), IFN-γ (1,000 U/ml), IL-4 (10 ng/ml) and IL-13 (10 ng/ml) were added to the wells in triplicate cultures. When used in combination TGF-β, IL-4 and IL-13 were used at 5 ng/ml each. Chemokine levels were determined in the supernatants by enzyme-linked immunosorbent assay upon 48 hours of further culture. The bars represent the mean ± standard error of eight distinct experiments in which SSc and control fibroblasts, matched for passage, age and sex of the donor, were cultured in parallel. IFN, interferon; IL, interleukin; IP, interferon-γ inducible protein; ND, not detectable; MCP, monocyte chemoattractant protein; SSc, systemic sclerosis; TGF, transforming growth factor; Th, T-helper.

In order to further explore possible differences between SSc and control fibroblasts, we assessed their capacity to produce chemokines in response to prototypic Th cytokines or to profibrotic TGF-β (Figure 2). None of the cytokines tested induced IL-8, but all of them (IFN-γ, IL-4, IL-13, TGF-β and combinations of IL-4 plus TGF-β and IL-13 plus TGF-β) induced MCP-1 production. Of interest, IFN-γ induced significantly higher levels of MCP-1 than did IL-4 or IL-13 (*P *< 0.005) and IL-4 or IL-13 higher levels than did TGF-β (*P *< 0.005). However, MCP-1 was synergistically induced by TGF-β added together with IL-4 or IL-13. Predictably, IFN-γ was a potent stimulus whereas all of the other cytokines inhibited IP-10 production. It is noteworthy that, although SSc fibroblasts tended to produce higher amounts of chemokines, the only statistically significant difference from control fibroblasts was the higher IP-10 production in the presence of IFN-γ (Figure 2).

### T cell membrane associated TNF-α, IL-1 and IFN-γ play distinct roles in inducing chemokine production by dermal fibroblasts

**Figure 3:**
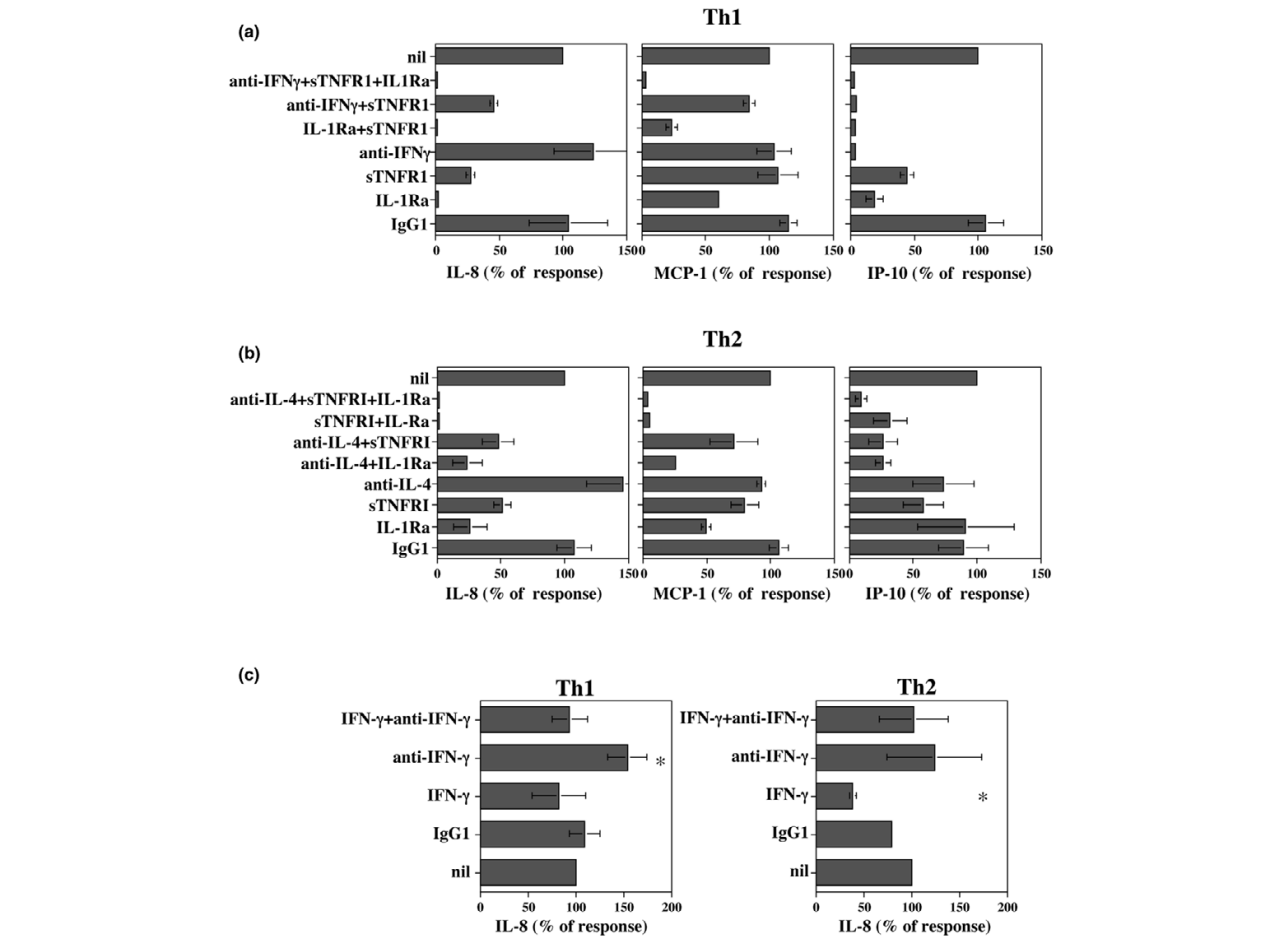
Differential effect of T cell membrane-associated cytokines in inducing IL-8, MCP-1, and IP-10. Fibroblasts were plated at 2 × 10^4 ^cells/well; 72 hours later the culture medium was replaced and T cell membranes corresponding to 2 × 10^5 ^cells generated from activated Th1 (panel a, *n *= 2; panel c, *n *= 4) and Th2 (panels b and c, *n *= 2) clones were added to the wells in triplicate cultures. Anti-TNF (soluble TNF receptor I; 10^-8 ^mol/l), IL-1 receptor antagonist (2 μg/ml), anti-IFN-γ (10 μg/ml), anti-IL-4 (10 μg/ml), irrelevant monoclonal IgG_1 _(10 μg/ml) and IFN-γ (1,000 U/ml), alone or in combination, were added to the wells 30 minutes before T cell membranes. Chemokine levels were determined in the supernatants by enzyme-linked immunosorbent assay upon 48 hours of further culture. The bars represent the mean percentage ± standard error of chemokine production in the presence of T cell membranes without blocking agent. **(a) **IL-8 was 8.8 ± 1.9 ng/ml, MCP-1 was 4.7 ± 0.2 ng/ml and IP-10 was 7.3 ± 1.1 ng/ml. **(b) **IL-8 was 36.5 ± 8.8 ng/ml, MCP-1 was 6.6 ± 0.9 ng/ml and IP-10 was 0.8 ± 0.3 ng/ml. Note that basal IL-8 levels were fourfold lower and IP-10 tenfold higher in the presence of Th1 compared to Th2 cells. **(c) **With Th1 cells IL-8 was 6.9 ± 0.7 ng/ml; with Th2 cells IL-8 was 12.0 ± 2.2 ng/ml. *P < 0.05. IFN, interferon; IL, interleukin; IP, interferon-γ inducible protein; MCP, monocyte chemoattractant protein; Th, T-helper.

In order to identify some of the mediators present in T cell membranes that induce chemokine production by fibroblasts, we used neutralizing reagents to block the biological activity of several cytokines. IL-8 induction was dependent on IL-1 and TNF-α, which exhibited additive effects in both Th1 and Th2 cells (Figure 3a,b). Because Th2 cells were more potent inducers of IL-8 production than were Th1 cells, we tested the hypothesis that IFN-γ could act as a partial inhibitor. Indeed, after exogenous addition of IFN-γ to fibroblasts activated by Th2 cells, IL-8 production was inhibited by more than 50%, and this inhibition was abrogated by IFN-γ neutralization (Figure 3c). Similarly, IFN-γ neutralization resulted in enhanced IL-8 production when fibroblasts were activated by Th1 cells (Figure 3c). MCP-1 induction by activated Th1 and Th2 cells was dependent on the synergistic effect of IL-1 and TNF-α. With Th1 cells, further reduction in MCP-1 production was observed when IFN-γ was neutralized (Figure 3a). Unsurprisingly, IP-10 induction by Th1 cells was mostly dependent on IFN-γ and, to a small extent, on TNF-α and IL-1, whereas the very poor IP-10 production induced by Th2 cells was essentially due to TNF-α with a marginal contribution from IL-1.

**Figure 4:**
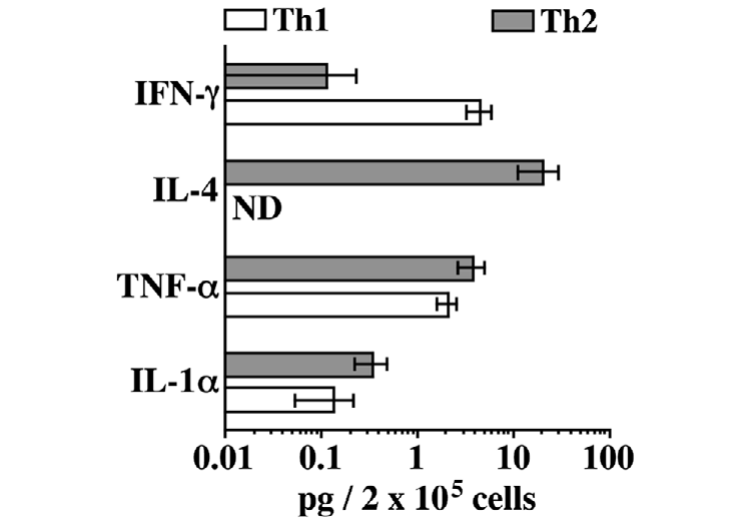
Cytokine content in the membranes of activated Th1 and Th2 cell clones. Cytokine levels were determined by enzyme-linked immunosorbent assay in Th cell membranes. Th cells were activated by CD3 cross-linking for six hours before membrane preparation. The bars represent the mean ± standard deviation of six Th1 and six Th2 cell clones, and indicate the cytokine levels in the cell membranes from 2 × 10^5 ^cells. When a given cytokine was not detectable, it was assigned the value 0 for mean determination. *P < 0.05. IFN, interferon; IL, interleukin; ND, not detectable; Th, T-helper; TNF, tumour necrosis factor.

We therefore attempted to verify whether IL-1α, TNF-α, IFN-γ and IL-4 could be detected in the T cell membranes of the clones used. This was indeed the case. IFN-γ was present in the membranes of all Th1 and in none but one of the Th2 cell membranes tested (Th1, *n *= 6; Th2, *n *= 6), IL-4 was present in none of the Th1 and all Th2 cell membranes tested, TNF-α was detectable in all membranes, and IL-1α was detectable in three out of six Th1 and in four out of six Th2 cell membranes tested (Figure 4). Thus, although IFN-γ and IL-4 were clearly mutually exclusive in polarized T cell membranes, this was not the case for TNF-α and IL-1α, which were present in both subsets.

**Figure 5:**
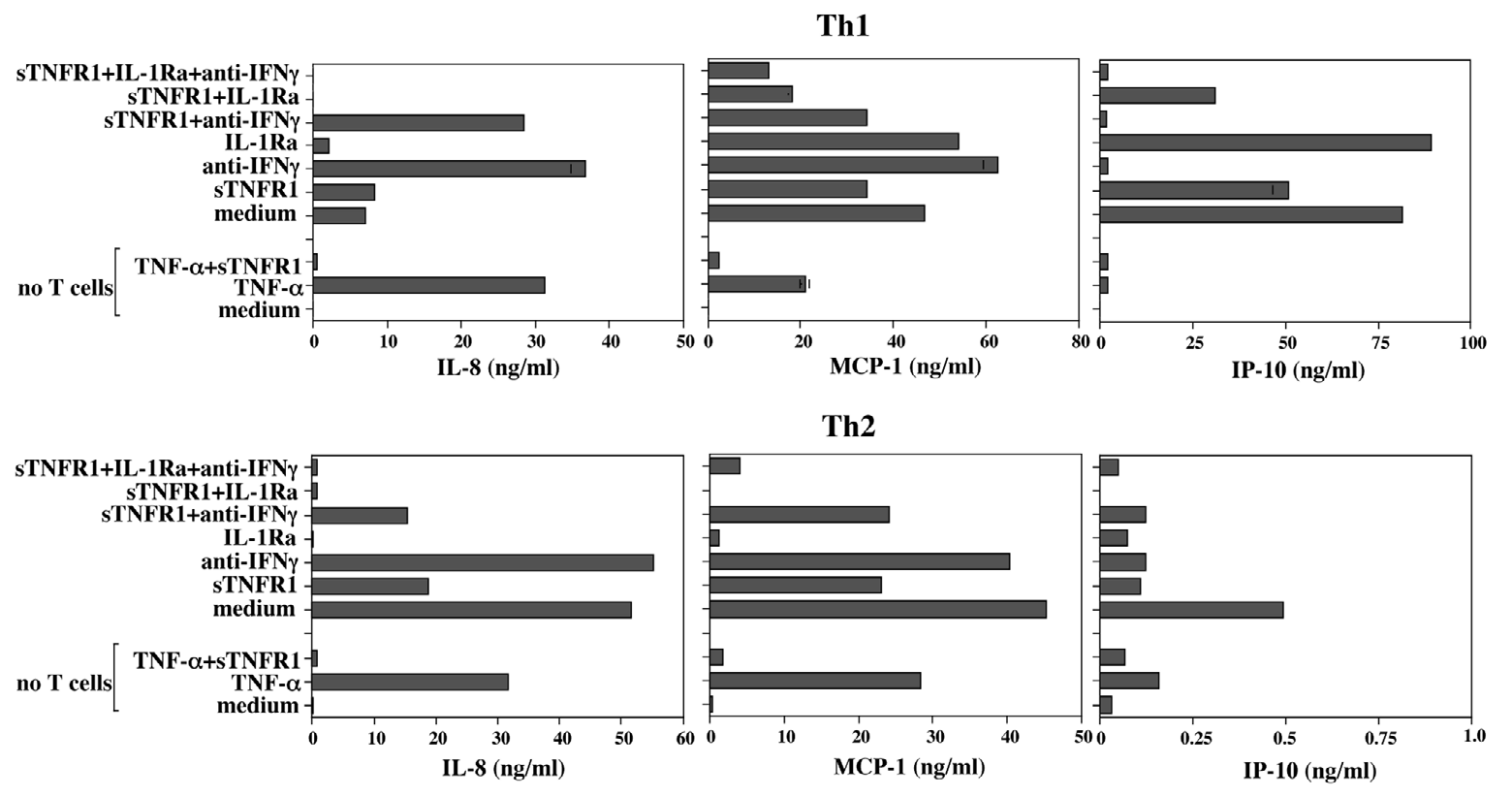
Living T-helper cells differentially induce IL-8, MCP-1, and IP-10 by fibroblasts cultured in transwell chambers. Fibroblasts were plated at 1.5 × 10^4 ^cells in the upper transwell chamber; 72 hours later the culture medium was replaced and T cells (1 × 10^6^) were plated in the lower transwell chamber previously coated with anti-CD3 mAb. Chemokine levels were determined by enzyme-linked immunosorbent assay in 48-hour supernatants. TNF-α (100 ng/ml) was used as a positive control in wells without T cells. Anti-TNF (soluble TNF receptor I; 10^-8 ^mol/l), IL-1 receptor antagonist (2 μg/ml) and anti-IFN-γ (10 μg/ml), alone or in combination, were added to the lower wells 30 minutes before T cells. The bars represent the mean of duplicate wells. Note that the scale is different in each panel and for IP-10 it is 100 times smaller for Th2 than for Th1 cells. Similar results were obtained in an additional experiment. IFN, interferon; IL, interleukin; IP, interferon-γ inducible protein; MCP, monocyte chemoattractant protein; Th, T-helper; TNF, tumour necrosis factor.

It has been reported that fibroblasts express CD40 and that they may be activated via interaction with CD40L (CD154) [3]. In our experimental conditions we could not observe any blocking effect of an anti-CD40L mAb when fibroblasts were cultured in the presence of T cell membranes whether from Th1 or Th2 clones, nor did we observe chemokine production by fibroblasts in response to recombinant human trimeric CD40L (not shown). We therefore tested whether fibroblasts would respond to cytokine produced by activated T cells in the absence of cell–cell contact. To this end, fibroblasts were cultured in the upper chamber of a semipermeable transwell, and living T cells were put in the lower chamber and activated by CD3 cross-linking. The results reinforce those observed when T cell membranes were used to activate fibroblasts (Figure 5). Fibroblasts exposed to cytokines produced by Th1 cells produced IL-8 and high levels of MCP-1 and IP-10, whereas those exposed to cytokines produced by Th2 cells produced high levels of IL-8 and MCP-1 but marginal amounts of IP-10. Furthermore, neutralization of TNF-α and IL-1, particularly when neutralizing reagents were used jointly, resulted in inhibition of induction of IL-8 and MCP-1 by Th1 and Th2 cells. In addition, neutralization of IFN-γ greatly enhanced IL-8 and abrogated IP-10 production induced by Th1 cells, with no effect on fibroblast responses induced by Th2 cells. Soluble TNF-α in the lower chamber, used as positive control, induced substantial amounts of IL-8 and MCP-1 but very little IP-10, and these effects were abrogated by its neutralization.

All together these findings indicate that TNF-α and IL-1, whether released in the supernatants or associated with T cell membranes, play a major role in inducing chemokine production by dermal fibroblasts. Differential effects observed with Th1 and Th2 cells are due to the role played by IFN-γ, which is produced only by Th1 cells, in that it inhibits IL-8 and stimulates IP-10 production.

### Intracellular signalling pathways mediating fibroblast activation by T cell contact

**Figure 6:**
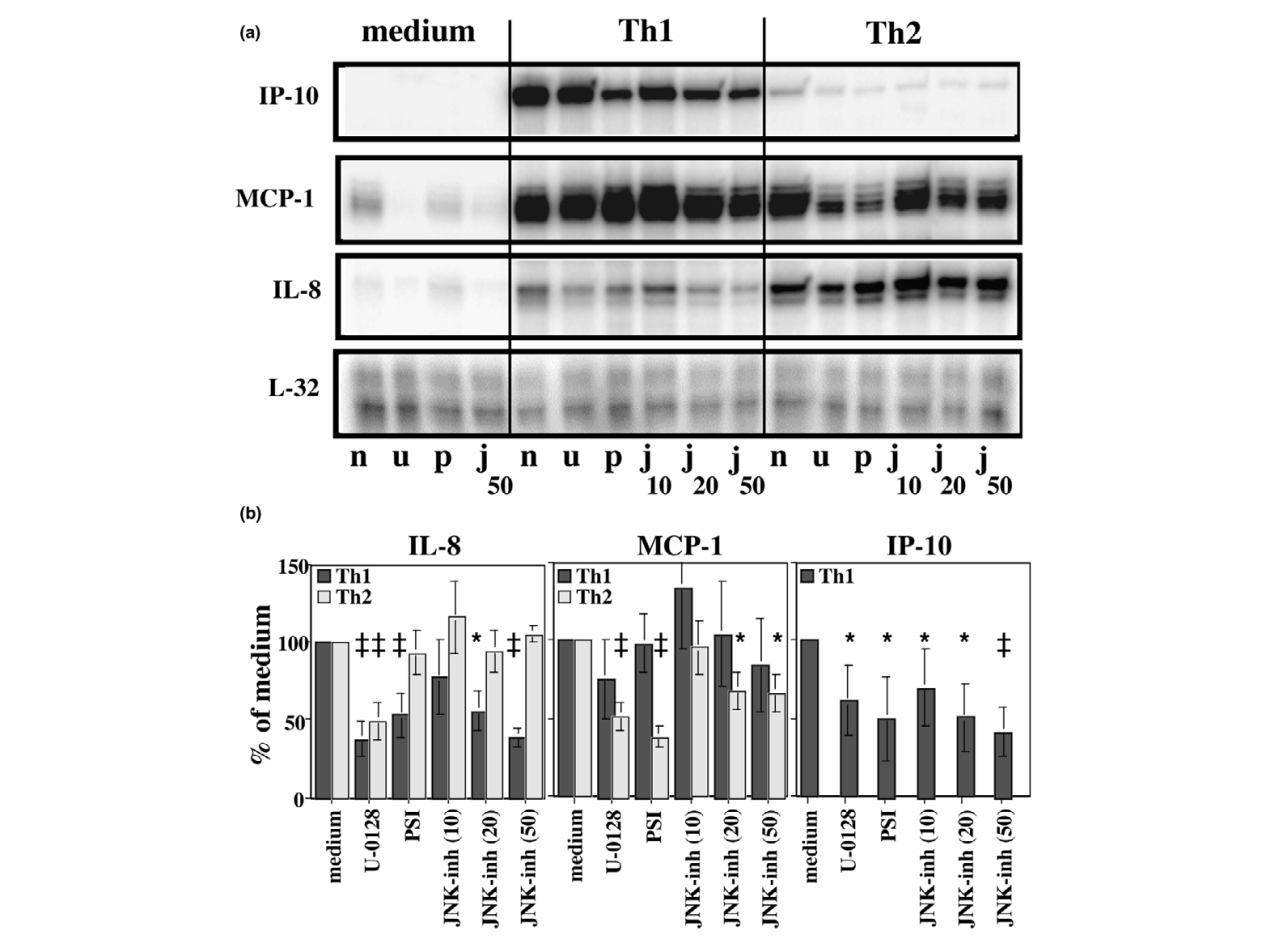
IL-8, MCP-1 and IP-10 mRNA in fibroblasts activated by T cell contact and effect of inhibitors. Fibroblasts were plated to confluence resulting in about 1 × 10^6 ^cells per Petri dish. They were serum starved overnight, and T cell membranes equivalent to 8 × 10^6 ^cells from Th1 and Th2 clones were then added for 4 hours in 1% FCS medium. Intracellular signalling inhibitors were added one hour before adding T cell membranes. Total fibroblast RNA was isolated and mRNA levels were assessed by RNase protection assay. **(a) **A representative analysis from five performed. n = nil; u = U-0126 (40 μmol/l); p = PSI (40 μmol/l); j = JNK inhibitor (10, 20 and 50 μmol/l). **(b) **mRNA signal intensity was determined densitometrically and normalized values, computed by dividing chemokine by housekeeping probe signals, were used to evaluate the effect of T cell contact and intracellular signal inhibitors. The bars represent the percentage of the chemokine signal intensity measured in the presence of T cell membranes with intracellular signal inhibitors divided by the signal obtained in the presence of T cell membranes without inhibitors. Shown is the mean ± standard deviation of five distinct experiments. Statistically significant differences versus medium: *P < 0.05, ^‡^*P *< 0.001. ERK, extracellular signal-regulated kinase; FCS, foetal calf serum; IFN, interferon; IL, interleukin; IP, interferon-γ inducible protein; JNK, c-Jun N-terminal kinase; MCP, monocyte chemoattractant protein; PSI, proteasome inhibitor I; Th, T-helper; TNF, tumour necrosis factor.

Consistent with the data obtained when evaluating protein production, the steady state levels of IL-8, MCP-1 and IP-10 mRNA were upregulated in fibroblasts activated by T cell contact, as compared with resting fibroblasts (Figure 6a). The relative intensity of the bands we observed was dependent on the T cell subset used to activate fibroblasts. Thus, IL-8 mRNA levels were higher and IP-10 mRNA levels lower in the presence of Th2 cells than in the presence of Th1 cells, whereas MCP-1 mRNA levels were similar (Figure 6a). To further document the capacity of polarized T cells to induce distinct patterns of chemokine production in dermal fibroblasts, we used pharmacological inhibitors in a series of five experiments in which we explored some intracellular signalling pathways used in response to T cell contact.

IL-8 mRNA levels were differently affected by the inhibitors tested. The inhibitor of the MEK/ERK pathway U-0126 strongly inhibited IL-8 mRNA induced by both Th1 and Th2 cells (Figure 6a,b). Inhibition of nuclear factor-κB (NF-κB) pathway by PSI and of JNK by SP600125 resulted in a selective and dose dependent (JNK inhibitor) decrease in IL-8 mRNA levels induced by Th1 but not by Th2 cells (Figure 6a,b). Of further interest, MCP-1 mRNA induced by Th1 cells was not significantly affected by any of the inhibitors used, but the MCP-1 levels induced by Th2 cells were affected strongly by both U-0126 and PSI and weakly by the JNK inhibitor (Figure 6a,c). Finally, IP-10 mRNA levels induced by Th1 cells were decreased by U-0126 and PSI, and dose-dependently by JNK inhibitor. Overall, these findings indicate that T cell contact triggers the simultaneous activation on fibroblasts of several intracellular signalling pathways and that their role in regulating chemokine transcription may vary according to the particular chemokine and subset of polarized T cells.

## Discussion

Emerging data suggest that chemokines may contribute to the development of fibrosis in SSc [49], a disease in which Th2-like responses dominate [33-36], although the presence of IFN-γ in lesional tissue has also been reported [34,50]. We specifically focused on the role of Th cell subsets in regulating the potential of chemokine production by dermal fibroblasts, because the interplay between these two cell types may be of particular relevance early in SSc development [32]. Several of the findings reported here are new. First, the pattern of chemokines produced by fibroblasts was shown to depend on the type of Th cell that stimulates them. This is consistent with the capacity of fibroblasts to activate flexible patterns of cytokine production readily. A corollary of this observation is the differential sensitivity to pharmacological inhibitors of intracellular signalling when fibroblasts were activated by Th1 and Th2 cells. Second, prototypic Th1 and Th2 cytokines did not induce on fibroblasts effects of the same quality and magnitude as those induced by polarized T cells. For instance, IL-8 was not induced by any of the Th1 and Th2 cytokines, but was strongly induced by Th2 and to a lesser extent by Th1 cells – an effect due to cytokines (IL-1 and TNF-α) shared by the two T cell subsets. This indicates that the biological activities of T cells do not simply mirror the cytokines that they preferentially produce and are used for classification purposes.

MCP-1 is highly represented in tissues undergoing fibrosis. Our findings indicate that fibroblasts are capable of upregulating MCP-1 production when they are exposed to T cell contact, and comparable amounts of MCP-1 are produced in response to either Th1 or Th2 cells. The results of blocking experiments indicate that membrane-bound IL-1 accounted for 50% of the MCP-1-inducing capacity of both subsets synergistically with TNF-α, whereas the effect of membrane-bound IFN-γ on Th1 cells was marginal. Of interest, IL-4, IL-13 and TGF-β inhibit MCP-1 production on macrophages and are in general considered suppressive cytokines [51]. In our experiments, soluble IFN-γ, IL-4 and IL-13, although with differential efficacy, induced substantial production of MCP-1 by fibroblasts. In agreement with our data, IL-4 has been shown to induce MCP-1 in lung fibroblasts [17] and endothelial cells [52], thus indicating that MCP-1 regulation is strictly dependent on cell type.

IL-8 is present in SSc skin and is produced by SSc fibroblasts in large amounts [53,54]. Consistent with the notion that IL-8 is induced by inflammatory cytokines, our neutralization experiments revealed that T cell membrane bound IL-1 and TNF-α were essential and sufficient inducers of massive IL-8 production by fibroblasts, whereas soluble Th1 and Th2 cytokines did not elicit IL-8 production. Of interest, IL-8 levels were significantly higher when fibroblasts were activated by Th2 than by Th1 cells. By assessing the effect of exogenous addition of IFN-γ and IFN-γ neutralization, we were able to demonstrate that IFN-γ, at least in part, accounts for the differential capacity of Th1 and Th2 in inducing IL-8. These results are consistent with the previously reported capacity of IFN-γ to suppress IL-8 production by fibroblast-like synoviocytes activated by inflammatory cytokines [55]. In fibroblasts, the transcriptional inhibition of IL-8 by IFN-γ was mediated by NF-κB [56]. Transcriptional inhibition has also been demonstrated in polymorphonuclear phagocytes [57]. However, interferons do not appear to be uniformly inhibitory to IL-8, because IFN-γ enhanced IL-8 gene expression by a post-transcriptional mechanism in monocytic cells [58], and had enhancing or inhibitory effects according to the timing of exposure on gingival fibroblasts activated by bacterial lipopolysaccharides [59]. Thus, the regulatory activity of IFN-γ on IL-8 may depend on subtle differences within a particular cell type and may vary depending on cell type.

In our experimental setting IP-10 was massively induced in fibroblasts by Th1 cells and, as expected, neutralization of membrane-bound IFN-γ totally inhibited IP-10 production. Of interest is that antagonists of IL-1 and TNF-α were also able to reduce IP-10 production substantially in response to Th1 cells. This is consistent with the reported capacity of these proinflammatory cytokines to enhance IP-10 production in cells that are poor IFN-γ responders [60]. However, the fact that IP-10 production by fibroblasts depends on IFN-γ is highlighted by the weak IP-10 response induced by Th2 cells. IP-10 has been detected in SSc serum at higher frequencies than in healthy individuals [61]. It should be stressed, however, that exogenous IP-10 administration in animal models of fibrotic diseases results in decreased fibrosis [30,31].

As a general feature, SSc fibroblasts compared with control fibroblasts exhibit higher spontaneous and stimulated ability to synthesize proteins, including MCP-1 and IL-8 [54,62]. In our experimental setting, we only observed that chemokine production by SSc fibroblasts tended to be higher, with IP-10 production induced by IFN-γ being significantly higher in SSc than in control fibroblasts. In this regard, it is important to point out that we used fibroblasts at early passages (from passages four to nine) and that, in parallel experiments, we found higher spontaneous collagen synthesis and enhanced resistance to inhibition by T cell contact using the same SSc and control fibroblasts [36].

The aim of our experiments in which pharmacological inhibitors of signal transduction pathways were used was to explore whether the effector molecules differentially expressed on the surface of polarized T cells would result in differential intracellular signalling. This proved to be the case, as is reflected by the differential efficacy of the inhibitors added in decreasing the steady state mRNA levels of IL-8, MCP-1 and IP-10 induced by Th1 and Th2 cells. Of particular interest was the finding that selective blockade of JNK resulted in major inhibition of IL-8 mRNA induced by Th1 but not by Th2 cells. Involvement of JNK in IL-8 induction has been demonstrated in other cell types [63,64]. In our system Th2 cells induced higher levels of IL-8 than did Th1 cells, an effect that is in part due to the inhibitory effect of IFN-γ present in Th1 cell membranes. Thus, it may be hypothesized that signals triggered in fibroblasts by IFN-γ rendered JNK a limiting signal transducer for IL-8 transcription. Contrary to the differential effect of JNK inhibition, blocking the MEK/ERK pathway of the mitogen-activated protein kinases resulted in IL-8 mRNA reduction on both Th1 and Th2 cells. This points to commonalities in signals initiated by membrane-associated molecules of both T cell subsets and involved in IL-8 induction (for example IL-1 and TNF-α). Furthermore, and consistent with our findings, IL-8 synthesis on fibroblast-like synoviocytes induced by bacterial products has been shown to depend both on ERK and JNK mediated signals [64].

As far as MCP-1 mRNA levels are concerned, a significant decrease was observed exclusively when Th2 cells were used to activate fibroblasts, whether in the presence of MEK/ERK, proteasome, or by JNK inhibitors. In different cell systems, NF-κB dependent signalling (targeted by the proteasome inhibitor we used) has been implicated in IL-1 and TNF-α induction of MCP-1 [65,66]. In our experimental system, T cell membrane associated IL-1 and TNF-α were identified as major inducers of MCP-1, with a contribution from IFN-γ in Th1 cells. The differential effects of signalling inhibitors used thus point to profound differences in the transduction signals triggered by polarized T cells, although the levels of MCP-1 mRNA and MCP-1 protein induced by Th1 and Th2 cells were similar.

Finally, proteasome and JNK inhibition resulted in a significant reduction in IP-10 mRNA levels induced by Th1 cells, whereas inhibition of MEK/ERK had only a marginal effect. We did not explore the contribution of STAT (signal transducer and activator of transcription)-1 signalling, which is the main pathway used by IFN-γ for IP-10 transcription. In agreement with our findings, based on the use of a proteasome inhibitor, NF-κB was previously implicated in IP-10 transcription when TNF-α cooperates with IFN-γ [67].

## Conclusion

We showed that T cells induce dermal fibroblasts to upregulate production of selected chemokines. The type of T cells – Th1 versus Th2 – determines the type of cytokines induced, and T cell effector molecules may be either membrane associated or released cytokines. Thus, IP-10 is produced preferentially by Th1 cells and IL-8 mainly by Th2 cells, whereas MCP-1 is induced equally by both subsets. This illustrates the capacity of fibroblasts to activate flexible patterns of chemokine production readily in response to the environment in which they operate, thus favouring different types of inflammatory responses. Of further interest is that flexibility in potential for chemokine production is maintained in fibroblasts derived from SSc skin.

## Abbreviations

CCL = CC chemokine ligand; CCR = CC chemokine receptor; DMEM = Dulbecco's modified Eagle medium; ERK = extracellular signal-regulated kinase; FCS = foetal calf serum; IFN = interferon; IL = interleukin; IP = IFN-γ inducible protein; JNK = c-Jun N-terminal kinase; mAb = monoclonal antibody; MCP = monocyte chemoattractant protein; MEK = mitogen-activated protein kinase kinase; MIP = macrophage inflammatory protein; NF-κB = nuclear factor-κB; PSI = proteasome inhibitor I; RANTES = regulated upon activation normal T cell expressed and secreted; SSc = systemic sclerosis; TGF = transforming growth factor; Th = T-helper; TNF = tumour necrosis factor.

## Competing interests

The authors declare that they have no competing interests.

## Authors' contributions

CC conceived the study, participated in its design and coordination, generated most of the T cell clones used, and drafted the manuscript. YP made substantial contributions to the acquisition of data (culture, enzyme-linked immunosorbent assay) and interpretation of data. AS performed skin biopsies and initiated fibroblast cultures. JMD was involved in revising the manuscript and provided important intellectual content. All authors read and approved the final manuscript.
